# Optical Coherence Tomography Biomarkers Predict the Long-Term Restorative Effect of Early Anti-VEGF Treatment on Diabetic Macular Edema

**DOI:** 10.3390/life15020269

**Published:** 2025-02-11

**Authors:** Süleyman Okudan, Sule Acar Duyan, Abdullah Erdem, Ayse Bozkurt Oflaz, Banu Turgut Ozturk

**Affiliations:** Faculty of Medicine, Department of Ophthalmology, Selçuk University, 42130 Konya, Türkiye; dr.sulenuracar@gmail.com (S.A.D.); erdemabd@gmail.com (A.E.); draysebozkurtoflaz@yahoo.com (A.B.O.); ozturkbanuturgut@yahoo.com (B.T.O.)

**Keywords:** anti-VEGF therapy, diabetic macular edema, diabetic retinopathy, optical coherence tomography biomarkers

## Abstract

Background/Purpose: This study compared the effects of three induction doses of anti-vascular endothelial growth factor (anti-VEGF) on diabetic macular edema (DME) with that of long-term treatment using biomarkers to find out the predictability potential of early response to anti-VEGF treatment for the long-term restorative effect. Methods: We retrospectively reviewed the clinical and optical coherence tomography (OCT) data of 71 DME eyes treated with three monthly anti-VEGF doses and followed for 1 year. BCVA, central subfield thickness (CST), subretinal fluid (SRF), intraretinal cysts, hyperreflective foci (HF), disorganization of inner retinal layers (DRILs), ellipsoid zone/external limiting membrane (EZ/ELM) integrity, and vitreoretinal relationships were assessed at baseline, 3, 6, and 12 months. Results: Patients (50.7% male) had a mean follow-up of 12 months. After three anti-VEGF doses, 19 eyes required no additional injections, 25 continued anti-VEGF, 20 switched to dexamethasone implants, and seven received combination therapy. Best corrected visual acuity (BCVA) improved from 0.52 to 0.40 logMAR at 3 months, 0.30 at 6 months, and stabilized at 0.40 at 12 months. CST decreased from 406 µm to 317 µm at 3 months and 307 µm at 12 months. Significant early improvements in BCVA, CST, SRF, and intraretinal cysts were sustained in the long-term follow-up. HF reduction became significant after 6 months, while DRIL and EZ/ELM integrity remained unchanged. Conclusions: The improvement of OCT biomarkers in DME patients supported that intravitreal anti-VEGF significantly restored the retinal microstructure, which was already evident at 3 months in the control after anti-VEGF induction.

## 1. Introduction

Diabetic macular edema (DME) affects approximately 21 million people worldwide and is the main cause of vision loss in diabetic patients [1]. The etiology and pathogenesis of macular edema are multifactorial. Through various inflammatory and vasogenic mediators, including vascular endothelial growth factor (VEGF) upregulation and inflammatory cytokines and chemokines, pathological changes in the vascular endothelium are induced and the blood–retinal barrier is disrupted. Therefore, fluid extravasation occurs in the extracellular space, manifesting clinically as macular edema and causing vision loss [2,3]. VEGFs have a pivotal role in the complex pathogenesis of DME, and anti-VEGF agents are currently the standard therapy [4]. Current DME treatment commence with three monthly initiation doses of intravitreal anti-VEGF injections and continues with anti-VEGF or steroids. Following the initial dose, the number of additional injections and the interval between injections are determined according to the response of the individual patient and the ophthalmologist’s preferred treatment regimen like PRN, treat and extend, etc. [5].

The response to treatment is quite variable in DME, and optical coherence tomography (OCT) has become the standard imaging modality used in the diagnosis and treatment follow-up of DME. It is able to quantitatively evaluate retinal thickness, volume, and all other morphological changes in retinal anatomy [6]. There are some biomarkers in OCT that are reported to be valuable in predicting prognosis and treatment response [7]. These include central subfield thickness (CST), subretinal fluid (SRF), intraretinal cysts, disorganization of the inner retinal layers (DRIL), hyperreflective foci (HF), ellipsoid zone (EZ), and outer limiting membrane (ELM) status [7,8].

Diabetic retinopathy is a leading cause of visual impairment worldwide, and the DRCR (Diabetic Retinopathy Clinical Research) Retina Network has been instrumental in developing treatment protocols, particularly through anti-VEGF therapies. These therapies have become a cornerstone in the treatment of diabetic macular edema (DME) and proliferative diabetic retinopathy (PDR) [9]. According to the DRCR retina network anti-VEGF treatment algorithm, the mean number of injections per year for a DME patient is greatest in year 1. It progressively decreases until year 5 according to the DRCR re-treatment algorithm [10]. Protocol I is a clinical trial conducted by the DRCR Retina Network and is an important milestone in the treatment of DME. It is one of the first large studies to evaluate the efficacy and safety of anti-VEGF therapy in diabetic macular edema. Protocol I reported the mean number of injections in DME patients as 8.1 in the first year, as 2.2 in the third year, and 1.9 in the fifth year. In the post hoc analysis of the Protocol I study, it was reported that the response after three anti-VEGF injections was preserved at 1 year and even at 3 years. It has been shown that deducing the long-term treatment response to anti-VEGF treatment is possible according to the outcome after three initial injections [11]. Though the number of injections is expected to lower with time, repeated injections usually become exhausting for DME patients after a period and raises questions about their benefits. Herein, we aimed to find out the effect of intravitreal treatment to regain vision and restore macular anatomy, and whether it is possible to predict the ultimate effect of anti-VEGF injections already following the three-monthly initiation dose. To reach this goal, the early responses of OCT biomarkers in DME patients are compared with those in the long-term response.

## 2. Materials and Methods

### 2.1. Study Design and Patients

This retrospective study included naive DME patients who received three initiation doses of monthly intravitreal anti-VEGF injections and were followed up for a minimum duration of 12 months. Patient files fulfilling this criterion were enrolled by scanning the registries of the Retina Unit between January 2018 and November 2023. The exclusion criteria were patients who underwent retinal laser photocoagulation for the treatment of diabetic retinopathy and those who underwent any other eye surgery procedure during 12 months. Additionally, patients with macular edema secondary to diseases other than diabetes and decreased visual acuity related to other causes like glaucoma, retinal detachment, macular hole, uveitis, optic neuropathy, or age-related macular degeneration were also excluded. The study protocol was approved by a clinical research ethics committee (Selçuk University Medical Faculty, 2024/650) and followed the tenets of the 1964 Helsinki Declaration.

Study parameters including age, gender, duration of DM, injections within 12 months, and best-corrected visual acuity (BCVA) (logMAR) were recorded from patients’ files. The OCT (DRI-OCT Triton; Topcon Corporation, Tokyo, Japan) images at baseline, after 3 monthly anti-VEGF doses, at the 6th month, and at the 12th month were graded for the parameters.

The switch to steroids has been decided upon the residual inflammatory OCT biomarkers after the initial 3 anti-VEGF treatments. Lens status and history of glaucoma have been considered as contraindications for dexamethasone implant application. Anti-VEGF molecule preference has been decided regarding the reimbursement regulations of the national social security department. Accordingly, it is mandatory to start with the 3 initial doses of bevacizumab. If necessary, further doses can be scheduled as aflibercept and ranibizumab if the patient presents a poor response.

### 2.2. OCT Measurements

Among the OCT parameters, central subfield thickness (CST), intraretinal cysts, subretinal fluid (SRF), hyperreflective foci (HF), disorganization of the inner retinal layers (DRIL), EZ/ELM status, and vitreomacular relationship were examined.

The size of the intraretinal cysts was graded using numbers as a reference, taking into account the size of the largest intraretinal cyst that could be identified on the scan. Cysts were classified as absent, mild, moderate, and severe [12].

All scans were divided into two groups (high HF/low HF) according to the mean number of HF using 30 as the cut-off value [12].

The absence of the EZ and/or the ELM was considered a complete loss of foveal reflectivity at this level, identified as the first and the second hyperreflective bands of the four outermost layers on OCT, respectively. These layers were defined as disrupted if they were not perfectly discernible but still partially visible in the fovea; if EZ was non-gradable due to the presence of SRF, ELM only was considered [12].

### 2.3. Statistical Analysis

The study data were analyzed in a computer environment with SPSS (Statistical Package for Social Sciences) 18.0 package program. In descriptive analyses, frequency data were shown as number (*n*) and percentage (%), and numerical data were shown using mean ± standard deviation or median value (minimum–maximum) according to the conformity of data to a normal distribution with the Kolmogorov–Smirnov test. The distribution of normally distributed numerical data in two independent groups was evaluated with the independent samples t-test, and the distribution of non-parametric distributed numerical data was evaluated with the Mann–Whitney U test. Mc Nemar and Mc Nemar Bowker tests were used to evaluate categorical data measured at baseline and T follow-up. The statistical significance level was accepted as *p* < 0.05 for all tests.

## 3. Results

The study included the 71 eyes of 71 DME patients. The demographic data and diabetes duration of the patients are shown in Table 1.

The evaluation of patient files revealed a baseline BCVA of 0.52 (0.22–0.82) logMAR and CST of 406.0 µm (324–478). Grading of the initial OCT images showed IRF in 98.6% of eyes, of which 54.9% were severe, 26.8% were moderate, and 16.9% were mild cysts. Only 1.4% of the eyes had no cysts. SRF was evident in 26.6% of enrolled eyes. Those with HF less than 30 constituted 64.8% of the eyes, while those with HF more than 30 constituted 35.2% of the eyes. DRIL was present in 67.6% of eyes. At baseline, EZ/ELM was intact in 50.7%, disrupted in 35.2%, and absent in 14.1% of eyes.

**Figure 1:**
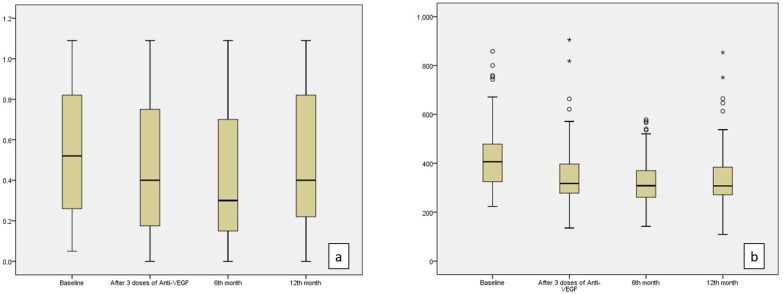
BCVA (logMAR) values (**a**) and CST (**b**) values demonstrated a statistically significant decrease at 3 months, 6 months, and 12 months compared to baseline values (*p* = 0.001, *p* < 0.001, *p* = 0.001 for BCVA and *p* < 0.001 for CST at the 3rd, 6th, and 12th months, respectively). (^o^; represents mild outliers, i.e., data points outside 1.5 to 3 times the interquartile range (IQR), but not extreme values. *; indicates extreme outliers, i.e., data points outside 3 times the IQR.)

The treatment results after three doses of monthly anti-VEGF therapy were analyzed in terms of treatment response according to the definition of refractory DME described as an increase in visual acuity (VA) ≤ 5 letters or a decrease in central subfield thickness (CST) ≤ 20% [13]. Accordingly, 46 eyes (64.8%) were labeled as responder and 25 eyes (35.2%) were labeled as non-responder to treatment and were recognized as refractory. After the third monthly visit, 19 eyes (26.8%) did not require additional injections, 25 eyes (35.2%) received additional anti-VEGF, 20 eyes (28.2%) received additional dexamethasone implants, and seven eyes (9.9%) received both anti-VEGF and dexamethasone implant treatment during 12 months follow-up. The initial three doses of intravitreal bevacizumab injection were performed. Of the eyes that received additional anti-VEGF injections, 15 received aflibercept, five received ranibizumab, and five continued with bevacizumab treatment. Among the eyes that received dexamethasone and anti-VEGF, three eyes received aflibercept and dexamethasone, three eyes received bevacizumab and dexamethasone, and one eye received ranibizumab and dexamethasone. The mean number of anti-VEGF injections per patient was 3.77 and 0.39 for dexamethasone during 12 months. The retreatment criteria were a loss of more than five letters or CST more than 250 µm. The BCVA improved after three doses of treatment compared to baseline (*p* = 0.001); this improvement continued at the sixth month (*p* < 0.001). At the final visit on month 12, it decreased slightly compared to the sixth month, but it was still significantly better than the baseline visit (*p* = 0.001) (Table 2, Figure 1a).

The second most frequently used parameter for DME treatment is CST in daily routine. The median value of CST was 406 µm at the baseline visit, which decreased to 317 µm after three doses of anti-VEGF. It demonstrated a statistically significant decrease to 308 µm at the sixth month and 307 µm at the 12th month (*p* < 0.001) (Table 2, Figure 1b).

**Figure 2:**
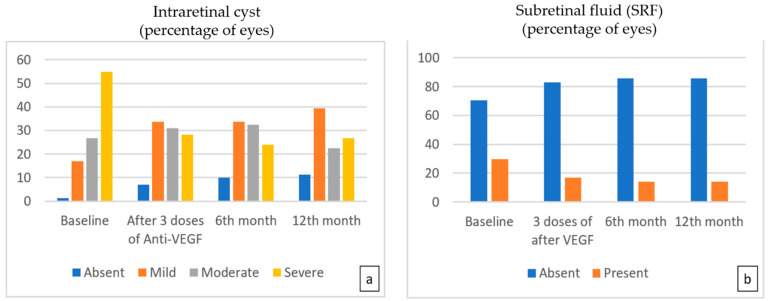
The percentage of eyes with moderate and severe intraretinal cysts (**a**) showed statistically significant improvement with time compared to baseline (*p* < 0.001 for the 3rd-, 6th-, and 12th-month visits, respectively). The percentage of eyes with SRF (**b**) presented a steady and statistically significant decrease during 12 monthly treatments (*p* = 0.022, *p* = 0.007, *p* = 0.007 for 3rd, 6th, and 12th months, respectively).

The evaluation of OCT biomarkers during the follow-up period showed that the number of eyes with severe intraretinal cysts decreased significantly after three doses of treatment and at the sixth and 12th months compared to baseline, while the number of eyes with mild cysts increased (*p* < 0.001). At the end of the 12th month, there was still IRF in 88.7% of eyes, but 44.4% of that was mild (Figure 2a). After three doses of treatment, SRF resolved in 42.9% of eyes, which was found to be statistically significant compared to the baseline percentage of 26.6% (*p* = 0.022). While SRF resolved in two more eyes at the sixth month, no further improvement was observed at the 12th month (Figure 2b) (Table 3).

**Figure 3:**
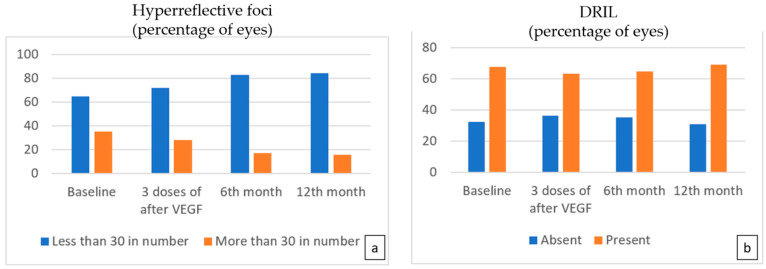
The number of eyes with more than 30 hyperreflective foci (**a**) decreased at each follow-up visit, but a statistically significant decrease was noted at the 6th and 12th months (*p* = 0.002, *p* < 0.001 for the 6th and 12th months, respectively). No significant difference in the number of eyes with DRIL (**b**) was found following treatment.

The change in the percentage of HF was slow during follow-up. Although there was a slight decrease in the number of eyes with more than 30 HF after three doses of anti-VEGF treatment, it was not statistically significant (*p* = 0.227); however, at the sixth-month and 12th-month visits, OCT images revealed a statistically significant decrease in the percentage of more than 30 HF compared to baseline (*p* = 0.002, *p* < 0.001) (Figure 3a).

There was no significant difference in the status of DRIL at the third, sixth, and twelfth months (Figure 3b).

**Figure 4:**
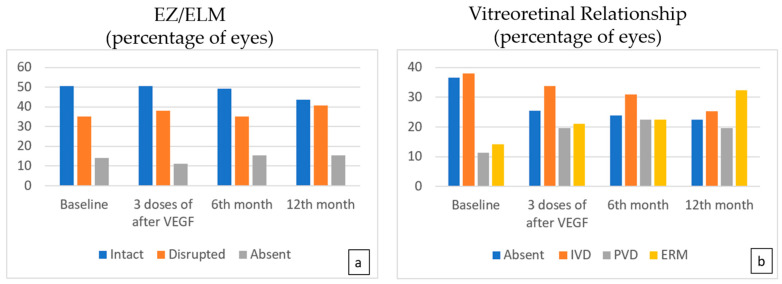
The EZ/ELM status (**a**) showed no significant difference following treatment. While a statistically significant decrease is observed in the number of eyes with IVD (**b**), an increase in the number of eyes with PVD was noted compared to the baseline (*p* = 0.023, *p* = 0.007, *p* = 0.001 at 3, 6, and 12 months, respectively).

At months 3, 6, and 12, there was no significant difference in the status of EZ/ELM (Figure 4a). The number of eyes with IVD was found to be lower at the sixth month compared to the baseline (*p* = 0.007) (Table 3, Figure 4b).

**Figure 5:**
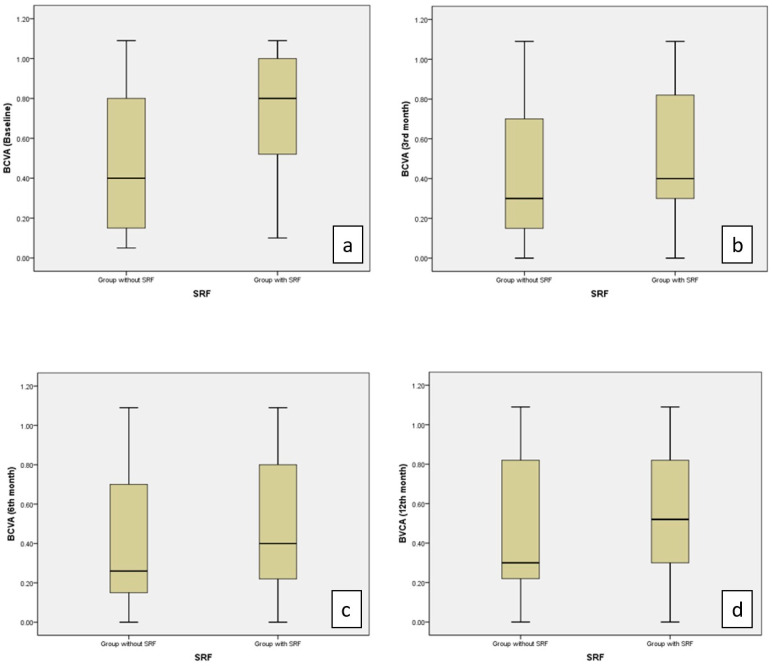
Values of BCVA (logMAR) were significantly higher in the group with SRF than in the group without SRF at the baseline (**a**) and at the 3rd month (**b**) (*p* = 0.012, *p* = 0.039, respectively). No significant difference was observed between the two groups in the 6th (**c**) and 12th months (**d**).

A comparison of BCVA at baseline and the third-month visits between patients with SRF and without SRF showed worse BCVA in the SRF group (*p* = 0.012, *p* = 0.039, respectively) (Table 4, Figure 5).

BCVA in the sixth month was found to be significantly worse in the group with a higher number of HFs at baseline compared to the group with a lower number of HFs (*p* = 0.015). When patients were divided into two groups in terms of DRIL existence, the mean BCVA was found to be worse in the group with DRIL at baseline, the third month, and sixth month (*p* = 0.047, *p* = 0.002, *p* < 0.001) (Table 5).

## 4. Discussion

The main objective of this retrospective observational study was to evaluate the response after three doses of monthly intravitreal anti-VEGF treatment and long-term response with OCT biomarkers in patients with DME.

The analysis of the data after three doses of monthly anti-VEGF treatment showed significant improvement in BCVA, CST, IRF, and SRF, while a significant decrease in HF occurred later at 6 months. The number of HF, intraretinal cysts, CST, and SRF was significantly reduced after intravitreal treatment, which suggested that the retinal microstructural integrity was pretty much restored by intravitreal agents.

In concordance with other previous studies, the improvement in VA was mostly observed in the third and sixth months of treatment, while VA remained more stable in the long term [4,14,15]. A similar study by Kriechbaum et al. [16] also reported that eyes treated with anti-VEGF therapy for DME showed a rapid improvement in VA in the first months of treatment, whereas VA improvement was slower in the long term.

Coscas et al. [17] first reported HFs as small punctiform hyper-reflective elements on SD-OCT. They suggested that HFs may represent aggregates of activated microglia cells. Vujosevic et al. [18] described HFs in the early stages of diabetic retinopathy (DR) and diabetic patients without DR. They also emphasized that these spots are mostly located in the inner retinal layers in the early stage and migrate toward the outer retinal layers as DR progresses. Previous studies have shown that HF, which is thought to have an inflammatory origin in DME, is significantly reduced after intravitreal treatment [19,20,21]. Vujosevic et al. [20] reported an early improvement in HF from the first month after anti-VEGF treatment. Schreur et al. [22] also showed a decrease in the number of HF after three monthly doses of anti-VEGF treatment. In our study, it was observed that there was no improvement in HF after three doses of anti-VEGF treatment, but there was a significant decrease at 6 months. While other OCT biomarkers such as CST, SRF, and intraretinal cysts responded earlier to treatment, HF responded later. In a study by Yoshitake et al. [23], it was highlighted that there was no improvement in the number of HFs in the first months after anti-VEGF treatment in eyes with DME, while there was a decrease in the number of HFs in the late period, as in our study. This late response might be explained by the longer duration of HF, but more research is needed to study the etiology and clinical significance of HF through histologic and epidemiologic studies.

CST is an important parameter for DME diagnosis and treatment decisions. The majority of published studies have shown that a reduction in CST is associated with an improvement in BCVA, but there are some studies reporting no direct correlation between BCVA and CST [8,21,24]. In our study, there was a statistically significant decrease in CST, as expected after three doses of anti-VEGF treatment and after six and twelve months.

Regarding SRF, the frequency of this biomarker in DME varies between 15% and 30% in different reports [25,26]. In this study, our results are consistent with the literature, as SRF was seen in 26.6% of the eyes at baseline. While the presence of SRF has been shown as a positive biomarker for better functional outcomes in some studies, there are also studies reporting the opposite. Vujosevic et al. [26] stated that the presence of SRF leads to ELM disruption and decreased retinal sensitivity. Seo et al. [27] reported that photoreceptors were more damaged in the presence of SRF and are therefore associated with poor visual outcomes. Moreover, they reported that DME eyes with SRF tended to respond poorly to anti-VEGF therapy. In contrast, another study reported that in DME patients, those with SRF had a better functional and anatomical response to anti-VEGF therapy than those without SRF [28].

In our study, the mean BCVA at baseline was worse in eyes with SRF. Although visual improvement was better in the group with SRF at the third and sixth month, it was not statistically significant.

Intraretinal cysts were another valuable OCT parameter investigated in our study. The pathophysiology of intraretinal cyst development is a combination of increased vascular permeability, leukostasis, inflammatory cytokines, VEGF, and Müller cell dysfunction [29]. In this study, there was a significant reduction in the number of patients with severe cysts in the early phase of treatment. Our observations that intraretinal cysts decreased during treatment with intravitreally injected anti-VEGF agents in patients with DME support the positive effect of anti-VEGF therapy on cystoid macular edema seen in previous studies [30].

DRIL is thought to indicate the disorganization or destruction of cells in the inner retinal layers and disruption of the visual pathways from photoreceptors to ganglion cells [31]. The International Retina Group demonstrated by OCT a reduction in DRIL extension after treatment of DME with dexamethasone, which they attributed to a favorable architectural effect on Muller cells due to a reduction in inflammation [32]. Vujosevic et al. [33] reported a significant decrease in DRIL extension with both dexamethasone and anti-VEGF treatment. In this study, no significant improvement was found in DRIL with anti-VEGF treatment. The mean baseline VA was worse in eyes with DRIL compared to eyes without DRIL, and no significant difference was observed in the BCVA of the DRIL-positive group with anti-VEGF treatment. The EZ/ELM status was also unresponsive to treatment; no significant improvement was observed with treatment. DRIL and EZ/ELM damage were mentioned as worse prognosis parameters, demonstrating limited improvement with treatment. Our results for these parameters are in accordance with the current literature [34].

In this study, patients who showed a poor response after three doses of anti-VEGF treatment were switched to anti-VEGF treatment and were switched to ranibizumab or aflibercept agents. In patients who showed a poor response and especially in those with prominent inflammatory biomarkers such as HF and SRF on OCT, treatment was switched to a dexamethasone implant. In patients who showed a poor response to anti-VEGF treatment, an early switch is important to prevent the permanent loss of retinal cells due to chronic edema [35].

The major limitation of this study was its retrospective nature and relatively small number of patients. Furthermore, the decision and timing of reinjection are variable. However, our results provide evidence from a real-world environment.

## 5. Conclusions

Our results revealed that intravitreal anti-VEGF treatment in DME resulted in a significant improvement of BCVA and OCT biomarkers including CST, SRF, and intraretinal cysts in the early phase. Hyperreflective foci known to be an inflammatory biomarker demonstrated a late response starting at 6 months. This improvement demonstrated that intravitreal treatment largely restored retinal microstructural integrity in DME starting up to three monthly initiation doses and provided the resolution of inflammatory findings in the long term.

## Figures and Tables

**Table 1:** Age, diabetes duration, and gender distribution of patients.

	Years
Mean age	63.80 ± 7.96
Mean duration of diabetes	14.12 ± 6.44
**Gender**	***n* (%)**
Male	36 (50.7)
Female	35 (49.3)

**Table 2:** Comparison of visual acuity and CST at baseline and after 3, 6, and 12 months.

	Baseline (n = 71) Median (1st–3rd Quartiles)	After 3 Doses of Anti-VEGF (n = 71) Median (1st–3rd Quartiles)	*p* Value	6th Month (n = 71) Median (1st–3rd Quartiles)	*p* Value	12th Month (n = 71) Median (1st–3rd Quartiles)	*p* Value
BCVA (logMAR)	0.52 (0.22–0.82)	0.40 (0.15–0.80)	0.001	0.30 (0.15–0.70)	<0.001	0.40 (0.22–0.82)	0.001
CST (µ)	406.00 (324.00–478.00)	317.00 (275.00–398.00)	<0.001	308.00 (259.00–372.00)	<0.001	307.00 (269.00–384.00)	<0.001

**Table 3:** Comparison of OCT biomarkers at baseline and after 3 doses of monthly anti-VEGF at 6th month and 12th month.

	Baseline (n = 71) n (%)	After 3 Doses of Anti-VEGF (n = 71) n (%)	*p* Value	6th Month (n = 71) n (%)	*p* Value	12th Month (n = 71) n (%)	*p* Value
Intraretinal cysts Absent Mild Moderate Severe	1 (1.4) 12 (16.9) * 19 (26.8) 39 (54.9) *	5 (7.0) 24 (33.8) 22 (31.0) 20 (28.2)	<0.001	7 (9.9) 24 (33.8) 23 (32.4) 17 (23.9)	<0.001	8 (11.3) 28 (39.4) 16 (22.5) 19 (26.8)	<0.001
SRF Absent Present	50 (70.4) 21 (29.6)	59 (83.1) 12 (16.9) *	0.022	61 (85.9) 10 (14.1) *	0.007	61 (85.9) 10 (14.1) *	0.007
Hyperreflective foci Less than 30 in number More than 30 in number	46 (64.8) 25 (35.2)	51 (71.8) 20 (28.2)	0.227	59 (83.1) 12 (16.9) *	0.002	60 (84.5) 11 (15.5) *	<0.001
DRIL Absent Present	23 (32.4) 48 (67.6)	26 (36.6) 45 (63.4)	0.375	25 (35.2) 46 (64.8)	0.625	22 (31.0) 49 (69.0)	0.999
EZ/ELM status Intact Disrupted Absent	36 (50.7) 25 (35.2) 10 (14.1)	36 (50.7) 27 (38.0) 8 (11.3)	0.506	35 (49.3) 25 (35.2) 11 (15.5)	0.842	31 (43.7) 29 (40.8) 11 (15.5)	0.076
Vitreoretinal relationship Absent IVD PVD ERM	26 (36.6) 27 (38.0) * 8 (11.3) 10 (14.1)	18 (25.4) 24 (33.8) 14 (19.7) 15 (21.1)	0.023	17 (23.9) 22 (31.0) 16 (22.5) 16 (22.5)	0.007	16 (22.5) 18 (25.3) 14 (19.7) 23 (32.4)	0.001

* refers to the group from which the difference originates. Third month, sixth month, and twelfth month compared with the baseline.

**Table 4:** Comparison of BCVA according to the presence of SRF.

	Group Without SRF (n = 50) Median (1st–3rd Quartiles)	Group with SRF (n = 21) Median (1st–3rd Quartiles)	*p* Value
BCVA (Baseline)	0.40 (0.15–0.80)	0.80 (0.46–1.00)	0.012
BCVA (3rd month)	0.30 (0.13–0.70)	0.40 (0.30–0.91)	0.039
BCVA (6th month)	0.26 (0.13–0.70)	0.40 (0.21–0.81)	0.128
BVCA (12th month)	0.30 (0.21–0.82)	0.52 (0.30–0.91)	0.403

**Table 5:** Comparison of VA according to the number of HFs and the presence of DRIL.

	Less than 30 HF (n = 46) Median (1st–3rd Quartiles)	More than 30 HF (n = 25) Median (1st–3rd Quartiles)	*p* Value	Without DRIL (n = 23) Mean ± SD	With DRIL (n = 48) Mean ± SD	*p* Value
BCVA (Baseline)	0.40 (0.20–0.82)	0.70 (0.35–0.96)	0.110	0.44 ± 0.36	0.61 ± 0.32	0.047
BCVA (3rd month)	0.30 (0.13–0.56)	0.70 (0.18–0.87)	0.058	0.27 ± 0.26	0.54 ± 0.34	0.002
BCVA (6th month)	0.22 (0.13–0.52)	0.70 (0.17–0.87)	0.015	0.21 ± 0.17	0.51 ± 0.34	<0.001
BCVA (12th month)	0.35 (0.20–0.70)	0.60 (0.30–1.00)	0.114	0.42 ± 0.32	0.54 ± 0.35	0.186

## Data Availability

Data are contained within the article.
